# *Jealousy Incarnate*: Quiet Ego, Competitive Desire, and the Fictional Intelligence of Long-Term Mating in a Romantic K-Drama

**DOI:** 10.3390/bs10090134

**Published:** 2020-09-03

**Authors:** Lorenza Lucchi Basili, Pier Luigi Sacco

**Affiliations:** 1 Independent Researcher, via Nazareth 2, 35128 Padova, Italy; lorenza.lucchi.basili@gmail.com; 2 Department of Humanities, IULM University, via Carlo Bo, 1, 20143 Milan, Italy; 3 metaLAB (at) Harvard, 42 Kirkland St, Cambridge, MA 02138, USA; 4 Bruno Kessler Foundation, via Santa Croce 77, 30122 Trento, Italy

**Keywords:** Tie-Up Theory, Tie-Up Cycle, romantic K-dramas, social cognition, long-term couple bonding

## Abstract

In this paper, we analyze a K-drama aired by the Korean TV network SBS in 2016, *Jealousy Incarnate*, as a case study of the application of the Tie-Up Theory to a romantic narrative as a form of simulation of human mating processes with social cognition valence. We find that this case provides us with an example of a mating process where the choice of the male partner by the female lead character does not privilege the one that should be preferable on the basis of the standard prediction of the experimental research on human mating. This discrepancy is a signal of a basic limitation of experimental research, that highlights the subjects’ preferences for abstract potential partners but is not able to fully account for the mechanisms that lead to the choice of a specific partner in a specific mating interaction. We argue that the narrative simulation viewpoint provides insights that are complementary to those of experimental research, and that a more comprehensive theoretical approach, such as the one offered by the Tie-Up Theory, may be helpful to account for both perspectives.

## 1. Introduction

How are long-term human couples formed, and why? This question, of fundamental importance to understand a central aspect of human existence, is at the root of a remarkable research effort, also due to the flourishing of experimental studies on human attitudes and behavior. Experimental research is driven by the search for common dispositional or behavioral traits in the largest, most statistically representative possible samples. As in many other fields of psychology and social science, also in the study of human mating the experimental approach has yielded important results, which are valid and robust even in an inter-cultural context [1]. Specifically, a significant sexual dimorphism has been found as to the most desirable characteristics of a long-term partner in the heterosexual couple [2], with men particularly emphasizing physical attractiveness and women especially caring for resourcefulness (wealth, social status, etcetera) [3,4].

Despite their robustness and intuitive appeal, these results reflect key aspects of experimental practice: Elicitation of preferences [5], search for hormonal signatures [6] in the abstract evaluation of partner characteristics [7], or in the reactions shown by experimental subjects toward models (generally, suitably chosen pictures or videos of human beings), be them real [8] or manipulated [9], being presented either in a laboratory treatment or in situations of ‘artificial’ human interaction such as speed dating sessions [10] or online meeting apps [11], or in hybrid contexts of observational reaction to the speed dating choices of someone else [12]. However accurate and methodologically rigorous, the experimental setting embeds subjects in an artificial choice context far from typical real-life interactions with possible partners. Experimentation in real-life contexts would be obviously unethical, and therefore the experimental setting is a viable second-best alternative for the analysis of human mating choices. Yet, an especially critical aspect of experimental research is the request to sample subjects to express their preferences for a generic partner, or for a specific partner in a minimal socio-relational setting (two situations that already differ significantly as to the relevant choice mechanisms [13]), whereas human romantic relations focus upon a specific partner in the context of repeated interaction, i.e., on an individual with largely unique characteristics, linked to the specific interaction history and evolution [14], possibly within a path of self-actualization and self-discovery [15]—a complexity that is hard to elicit experimentally and even more to observe directly in real-life situations. Consequently, whereas experimental research typically addresses questions such as “what kind of partner would you be willing to mate with?”, the implicit question in real-life interactions is rather “would you be willing to mate with this specific dating partner?”. These are pretty different questions, both of which relevant for mechanisms that govern human mating processes, and that need to be considered in their interdependence. Only looking at the ‘social’ aspects of desirability standards or at the contextual aspects of real-life interactions, without accounting for their reciprocal influence, may be misleading.

As to scientific methods that provide generalizable results, the only solid option seems therefore that of working on large, representative samples to discover tendencies that apply to wider populations. However, the question of how and why human couples are formed or broken has not only scientific, but also social and economic interest, and human cultures have spontaneously developed what could be considered a complementary approach to the experimental one, that does not focus on general laws but on the interaction between, and on the choices of, specific (though imaginary) human subjects: Fictional narratives [16].

Nothing seems to be less scientifically compelling than a fiction for the interpretation and explanation of social facts. Not only ‘stories’ deal with a small (and thus statistically un-representative) bunch of characters, but such characters are not even real but imagined. Not only the ‘experimental’ sample is small, it is also arbitrary as subjects have not been selected but literally made up, and then shaped at will to let the story go wherever one likes—in an experimental setting, this would be outright fraud. This is nothing but the antithesis of what a scientific experiment looks like. 

However, this contraposition moves from a deep misunderstanding of the socio-cognitive valence of fiction, which presents in fact significant analogies with scientific modeling [17]. Cognitive psychology [18], humanistic psychology [19], and literary studies [20] are generating research programs that take the adaptive value of fiction seriously. Such programs characterize fictional narratives as sophisticated forms of social simulation that extend the experience base of individuals much beyond its lived dimension [21], enhancing the capacity of adaptive response to unforeseen or unfamiliar situations and circumstances [22], and providing a rationale for the intense, widely documented human craving for fiction [23], whose strength and universality hints at the possibility of an epigenetic drive [24]. If a fiction reaches a certain level of social validation, it assumes a super-factual valence, acquiring a bigger evidential weight than that of facts themselves [25] and offering valuable insights into the complexities and contradictions of human experience [26]. In this perspective, socially validated romantic narratives, if charged with such super-factual valence, become of interest in our inquiry on human mating [27], as they enable us to carry out ‘simulation’ analyses of whether a certain type of interaction between two specific, properly chosen fictional subjects leads or not to a couple with certain characteristics, instantiating that complementary viewpoint that remains inaccessible through the experimental approach [28]. The key point then becomes the social validation mechanism that makes certain fictions salient and noteworthy as sources of learning and reflection not only for researchers, but for all humans. The fact that certain fictions are inter-generationally transmitted and often drawing interest from audiences that are socio-culturally remote from the narrative’s native one, provides the most direct and reliable observational correlative of this validation process [29]. Such validation is not about accuracy of the narrative’s historical reconstruction nor about the plausibility of its situations and events—not incidentally, we can have socially validated stories that refer to entirely implausible or apparently imagined environments in physical, technological, or social-historical terms (as in science fiction, fantasy, or fairy tales), and that nevertheless maintain their meaningfulness in time and space, sometimes over the course of many generations [30]. What is being validated is the psychological plausibility of the fictional characters, that need to be credible enough to elicit identification in the audience, or grab attention and acquire widely acknowledged salience value (possibly a negative one; e.g., [31]) and memorability, as confirmed by the inclusion of the fiction within a socially significant narrative corpus [32].

Analyzing fictions as complementary knowledge sources to experimental data [33] yields different insights than those of mainstream analyses. The mechanisms that govern the interaction with a specific partner in a specific situation are not necessarily antithetical to those found in experimental analysis, but highlight elements that failed to emerge otherwise, and that call for a more comprehensive theoretical framework. An instance of such a framework is offered by the Tie-Up Theory [28,34,35], a new approach to the analysis of human mating centered upon the interaction between specific subjects, but also accounting for the social influences that reflect the general tendencies found in experimental research. Such theory may generate claims that can be experimentally tested, as well as claims that can be tested in the simulation lab of socially validated fictions. This latter line of research has been developed so far with reference to some of the most popular Hollywood romantic comedies of the last decades [28,36], and to the main romantic fairy tales [37]. Such preliminary research shows that the reasons that move a subject to choose a specific partner among many may also be independent of, and sometimes in contrast with, the characteristics attached to an ‘ideal’ partner via the abstract preferences possibly expressed by that same subject in an experimental setting, by evaluating pictures of human subjects or hypothetical situations, or being exposed to artificial and minimal forms of relationality. 

The romantic fictions that have drawn the most attention and become memorable, owe this salience to their insightfulness into situations or topics of particular interest and complexity, providing adaptively valuable information despite their fictional nature and the reference to specific and possibly implausible situations. Fictional repertoires may be a treasure trove of refined wisdom on a wide range of mating-related issues, including paradoxical or particularly complicated ones. Fictions can then be considered a sort of ‘social calculus’ condensed into a story, often crystallized through the layering of variants and additions—a calculus that through suitable analytical tools reveals explicitly the informational content that in its normal functioning mode is absorbed and transmitted through the subtle mechanisms of social cognition [38]. 

In this paper, we analyze, through the framework of the Tie-Up Theory, a relatively uncommon case: That of the abundance in the choice of desirable potential partners. If generally finding a desirable partner is difficult also due to intense sexual competition, what happens when competition is between choice options instead? Specifically, we address the following question: Can a woman love two men at once? The answer to this question is more nuanced than one could think, and through a suitably chosen narrative ‘simulation’ we will see how the outcome of this mating dilemma may lead the woman to choose the potential partner who would be likely turned down according to the abstract criteria that emerge from the experimental literature. The chosen fiction belongs to an especially interesting corpus: That of K-dramas, the South-Korean TV mini-series that have now become a global cultural trend [39], attracting and engaging audiences worldwide [40]. Romantic K-dramas are very popular and widely followed both on TV and on online streaming platforms [41]. Their abundant production obviously greatly varies in quality and audience appreciation, but the most popular series, also thanks to the impressive global public they manage to reach, are exposed to very selective and rapid inter-cultural social validation tests [42]. In this paper, we consider a successful 2016 series that offers an interesting answer to the question posed above. Rather than covering the entire narrative arc of the drama, we focus upon a particular scene that provides us with a meaningful answer to our question, and casts light on the interaction between the relatively more subjective vs. social factors in determining the choice of the partner in the context of Tie-Up Theory. Our result cannot be taken of course as a general answer to the question but rather, being a simulation analysis, as an explanation of how and why, in certain circumstances, a person’s actual choice of the romantic partner may be so different from the one expected from the theoretical predictions in terms of abstract desirability and adaptive value within the bio-socio-evolutionary economy of the couple.

The remainder of the paper is organized as follows. Section 2 contains a short synthesis of the key concepts of Tie-Up Theory. Section 3 considers K-dramas as a research area of special interest for romantic fictions and introduces the case study, whose analysis is presented in Section 4. Section 5 offers a short final discussion.

## 2. Methods I. Tie-Up Theory: Basic Concepts

In this section, we present, in a necessarily compact, schematic form, some of the key concepts of Tie-Up Theory. For a more complete presentation see [28,34,35].

**Figure 1:**
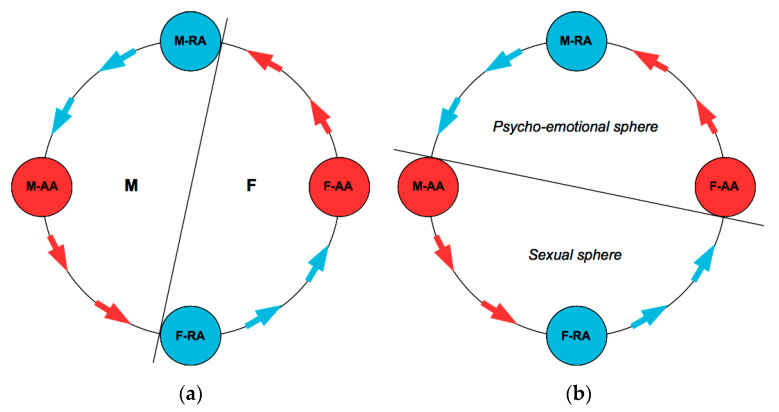
Tie-Up Cycle (TU-C) diagram and its possible partitions into hemicycles: (**a**) According to sex (male vs. female); (**b**) according to dimension (psycho-emotional vs. sexual). Red denotes Active Areas (AAs) and their provision of direct rewards, blue denotes Receptive Areas (Ras) and their provision of indirect rewards. The arrows show the natural direction of the cycle.

The Tie-Up Theory postulates that the human mating process is regulated by the interaction of two areas, the Active Area (AA) and the Receptive Area (RA) for each male (M) and female (F) subject. AAs function at the conscious level and are sensitive to social influence factors, whereas RAs typically function at the sub-conscious level and their activation is linked to the outcome of a specific Compatibility Test on the opposite-sex subject. Areas have a sexually dimorphic nature: The female AA (F-AA) responds to the psycho-emotional dimension, whereas her RA (F-RA) responds to the sexual dimension; for males, vice versa, M-AA responds to the sexual dimension and M-RA to the psycho-emotional one. Consequently, the Compatibility Test carried out by F-RA checks for the biological compatibility of the male subject, while the test carried out by M-RA checks for psycho-emotional compatibility of the female subject. Insofar as the opposite-sex subject passes the respective Compatibility Test, the involved RA turns on and prompts the subject to interact with the tested opposite-sex subject in view of a possible mating. Such interaction is structured according to a typical sequence known as Tie-Up Cycle (TU-C), that is the sequential stimulation of the AAs and RAs of the two subjects following a characteristic order. The TU-C may move from any position provided that order is respected: For instance, starting from a subject’s RA, the TU-C moves toward the same subject’s AA, which in turn stimulates the opposite-sex subject’s RA, further proceeding toward the respective AA, which will in turn stimulate again the first subject’s RA, closing the circle and paving the way to new iterations. The stimulation of the Areas produces specific rewards, that we call direct if generated by AAs and indirect if coming from RAs. When the sequence of interactions proceeds according to the foreseen order, the TU-C moves anti-clockwise. In some cases, the direction of movement may undergo a partial inversion due to frustrations enforced by one partner over the other [34]. The TU-C may be divided into hemicycles on the basis of two different criteria: The male (including M-AA and M-RA) vs. female (including F-AA and F-RA) hemicycle, and the sexual (including M-AA and F-RA) vs. psycho-emotional (including M-RA and F-AA) hemicycle (Figure 1). The (sexual or psycho-emotional) orientation of the respective Areas reflects specific evolutionary incentives that enable a couple with a functioning TU-C to stabilize into a cooperative interaction aimed at strengthening the couple bond and allowing the joint rearing of the offspring [35]. 

If the interaction between M and F leads for both to the production of the suitable direct and indirect rewards, the TU-C starts. The respective RAs of one or both subjects may have already carried out their Compatibility Tests or not, but even if this has not occurred yet, as the TU-C (and thus the interaction) unfolds, the test will end up being carried out. If a subject passes the Compatibility Test, the opposite-sex subject’s RA turns on, starting to produce indirect rewards. If and when such indirect rewards reach an intense enough peak, a Tie-Up (TU) will be created with the subject who passed the test. The TU may be one-sided if occurring only in the female (F-TU) or male (M-TU) subject, or it may be double and thus reciprocal (D-TU). A couple may be formed for various reasons also in the absence of a D-TU, and vice versa the emergence of a D-TU does not guarantee that the couple will be formed, but if the D-TU is created, the couple is formed, and the TU-C is successfully iterated, the resulting couple will be characterized by remarkable stability and resilience features.

The TU essentially depends upon the activation of RAs, but the role of AAs is equally crucial, in that the action of the latter may amplify or oppose the signals coming from the RAs. Specifically, being M-RA psycho-emotionally oriented, it follows that M-TU calls for a test of psycho-emotional compatibility, whereas being F-RA sexually oriented, F-TU requires that bio-sexual compatibility is tested. Vice versa, men are especially sensitive to social signals in the sexual sphere (given the orientation of the M-AA) while women are sensitive to social signals in the psycho-emotional sphere (due to the orientation of the F-AA). The ample experimental evidence showing that men pay special attention to the female partner’s physical aspect whereas women focus upon the social resourcefulness of the male partner then reflects the orientation of the respective AAs, and not of their RAs, and the reason why such orientations emerge so powerfully from experimental elicitations is that they address the conscious level of experimental subjects, that is their AAs. On the other hand, the (direct) sexual reward for men and the (direct) psycho-emotional reward for women, however pleasant and actively sought, are never enough by themselves to guarantee the TU, and may even end up precluding it, if not accompanied by the respective indirect rewards, with their different nature in each sex. Despite the declared preferences, therefore, a man will not tie-up to a female subject if he will not find a psycho-emotional compatibility, and a woman will not tie-up to a male subject if she will not find a bio-sexual compatibility. Of course, a couple may form also in the absence of TUs if driven by opportunistic or manipulative motives or if abiding by certain social norms (such as those regulating convenience marriages in some societies).

**Figure 2:**
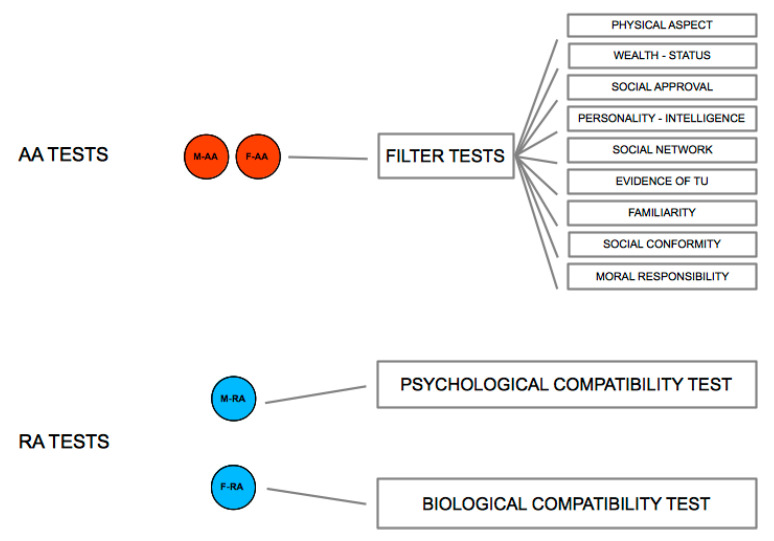
Tests carried out by the AAs and RAs on potential opposite-sex partners. Active Areas are in red, Receptive Areas in blue.

**Figure 3:**
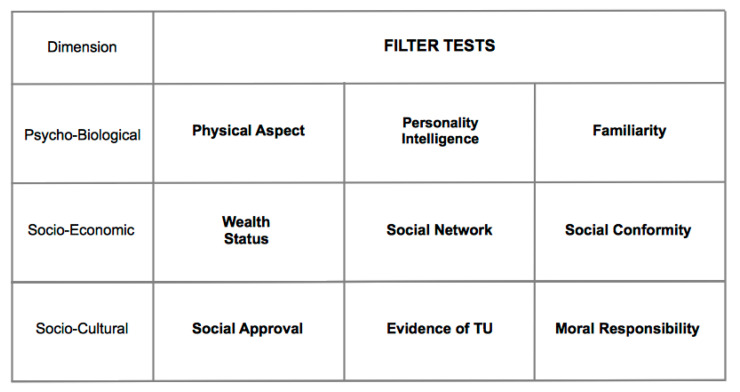
Different categories of Filter Tests.

Not only the RAs, but also the AAs carry out their tests on opposite-sex partners, which however refer to social appropriateness criteria and are not sexually dimorphic (Figure 2). Such tests, called Filter Tests, may vary according to the socio-cultural context of reference. Some of the main Filter Tests are shown in Figure 3. When the outcome of Filter and Compatibility Tests is concordant, all the incentives work toward the consolidation or the disruption of the couple (depending on whether the outcomes concordance is positive or negative). However, when outcomes are discordant, such discordance brings about inner conflicts in the subjects, which in turn cause conflict between the subjects, and even possibly synergize with operating social conditionings. The successful or failed formation of the couple thus depends on the extent to which the interaction between the subjects will create the conditions for the resolution or mitigation of the conflicts, or rather will make them deflagrate. Depending on the configuration of the outcomes of the Compatibility and Filter Tests, different types of couples may emerge, most of which are not characterized by a D-TU, and whose stability, even temporary, may depend on the action of a multitude of factors, as described by the Mating Stability Matrix [35].

## 3. Methods II. Romantic K-Dramas: A Promising Research Field for the Narrative Analysis of Human Mating

South Korea stands out as an emerging country in the global geography of creative production of the last two decades, a phenomenon known as Hallyu (literally: Korean wave [43]). Although Hallyu covers diverse sectors such as music, cinema, food and fashion [44], TV series (K-dramas) are especially important as they have attracted a global audience [45], and sparked an unprecedented interest toward Korean culture, and toward Seoul as a global cultural city and destination [46]. Romantic K-dramas, more specifically, deserve attention as products of a culture that, despite having been historically marked by extreme gender inequality [47], is now developing new narratives driven by new, vocal female agency [48], also thanks to the country’s high level of digital literacy that turns online platforms into spaces of co-creation where TV producers can acquire detailed feedback from viewers to take advantage of in future projects. Moreover, as most K-dramas are shot while the series is being broadcast (the so-called live-shoot system) [49], it becomes possible to use the feedback received on the early episodes to directly edit the screenwriting of the later ones on-the-go [50]. In addition to audience ratings, the K-dramas industry also considers involvement of audiences as to number of published articles, volume of online searches, intensity of engagement on discussion boards, and so on (the so-called Contents Power Index, CPI). This co-creation process has brought about a change in the themes and contents of romantic K-dramas which, responding to the indications and expectations of their (not only) young female public, explore the subtleties of romantic relationships also as a form of emancipation from the social constraints of traditional Asian cultures, such as arranged marriages [51], and are winning an increasingly intercultural audience, both in the Far East [52] and globally [53].

What makes K-dramas so interesting as social simulations of the dynamics of mating is the transitional state of Korean society with respect to so many highly relevant aspects of mating interactions, such as gender discrimination vs. equality [54], family control vs. personal autonomy [55], customary roles vs. intimacy in romantic relationships [56], and so on. This state of flux leads to a complex interplay between the declining (but still relevant) influence of parental, family, and social conditionings (on issues such as reproductive rights [57] or marriage decisions [58]), and the increasing but often unfulfilled drive toward search for authenticity and self-realization in mating choices (significantly, in the crucial socio-cultural transitional phase of the early 2000s South Korea was characterized by a particularly high relative frequency of divorce as compared to other Asian countries [59]). In turn, this very socially relevant tension is extensively documented in K-drama productions and, in their best examples, especially in the last 15 years, reflects into screenplays that offer an insightful, analytically detailed exploration of the dynamics of couple formation, and of their complications and contradictions [60]. Such features respond to K-drama audiences’ demands for cognitive insights and socio-behavioral guidelines to navigate the still relatively uncharted territory of autonomic styles of romantic relationships in societies with a strong traditionalist and patriarchal imprint [61].

This social cognition valence also partly explains the extraordinary success of K-dramas in geo-cultural environments out of South Korea, which are experiencing similar although often less mature social transitions, such as many South-East Asian [62], Muslim [63], or South American [64] countries—a success that cannot be merely explained in terms of the lure of romantic daydreaming for young female audiences [65]. Such a reductive, simplistic explanation stems from an erroneous maintained equivalence between K-dramas and soap-operas [66]. In soap operas, the intelligence of romantic relationships is typically entirely sacrificed to keep audiences engaged along an over-inflated, repetitive narrative going on for hundreds of episodes. In soap operas there is little concern for the psychological plausibility of the characters, or for the logical consequentiality of the narratives. The poor social cognition content of these narratives possibly leads viewers to develop dysfunctional beliefs and expectations about romantic relationships [67]. K-dramas generally are, to the contrary, mini-series of 16–24 one-hour episodes where, again in best examples, the narrative arc strives for compactness, plausibility, and coherence, and is carefully scrutinized and discussed in depth by fans [68]. K-dramas are an important, bi-directional global platform for cultural exchange and innovation and for social critique [69], whose audience contains well-educated, media-savvy, digitally sophisticated fandom circles [70]—a far cry from the typical content spectra and audience targets of soap operas (which exist in the current landscape of Korean TV production as well). Also in view of the relative sophistication of their audiences, generally high-quality romantic K-dramas obtain good viewer ratings and CPIs, although a romantic K-drama may be, at least in the short-term, successful and engaging also irrespectively of its social cognition content.

In the perspective of the Tie-Up Theory, K-dramas offer a rich repertoire (among many possible others) of narrative simulations of the variety of tied-up vs. opportunistic and dysfunctional couples that may emerge from romantic interactions under different circumstances, for instance by focusing on the role of aspirational parents and social expectations in framing marriage as a strategic assortative mating, typically on an educational and/or income basis [71,72]. As discussed above, Active Areas are sensitive to social pressures, so that parental, family, and reference group conditionings often reflect into the nature and outcomes of the main Filter Tests to be carried out. However, the main characters of K-dramas often fight or turn upside down the logic of more socially imposing Filter Tests, in their pursuit of their own path of personal autonomy and romantic authenticity, whereas, tellingly, such logic tends to be embraced by the antagonists seeking opportunistic forms of mating. In this sense, K-dramas offer a particularly valuable simulation analysis of mating interactions for all those socio-cultural contexts where the locally salient Filter Tests reflect social forces and constraints that are close enough, also by way of cultural analogies, to those of the Korean society of today [73]. However, RAs are shaped by deeper, very-long-term processes of biobehavioral programming, and their reactions tend to be less culture-specific and more universal. Therefore, K-dramas provide valuable insights in regard to the conditions for the emergence and stability of TUs in the context of romantic relationships also to cultures whose mating-related social conditionings and issues are of different nature. For all these reasons, K-dramas offer a particularly interesting knowledge base for research on narrative simulations of mating processes, and deserve to be further dug up in this vein from multiple disciplinary perspectives, such as communication and media studies, sociology of culture, and social and personality psychology.

It is this peculiar cultural dynamism that makes romantic K-dramas so intriguing, at least in their most significant, socially validated expressions. Here, we consider an interesting case study: The K-drama *Jealousy Incarnate*, aired in 2016 by the Korean national network SBS with a very good audience response, as testified by the high ratings in a difficult broadcasting slot such as the Wed–Thu 10 PM one, winning five SBS drama awards for categories closely related to the characters’ popularity: Top Excellence Actress for the female lead, Top 10 Stars Award and Top Excellence Actor for the male lead, and New Star Awards and Special Actress for the two co-leads (the male and female antagonists, respectively; source: koreandrama.org).

As we will see, this K-drama is a representative example of a romantic fiction with a high mating-related social cognition valence, tackling a complex issue such as the female choice between two potential male partners who both passed the Bio-sexual Compatibility Test, and characterizing with precision and consistency the interaction between the functioning of the Compatibility and Filter Tests, yielding an outcome that contradicts the likely prediction supported by the experimental literature. The interest of *Jealousy incarnate* does not stem from dealing with issues of social conditioning or parental manipulation, but is rather due to its focus on the fine-grained relational dynamic between the characters’ RAs and AAs, and represents therefore a case study whose ‘universal’ implications prevail upon culturally specific ones. The story’s resolution crucially revolves around a Filter Test, which is not culturally specific but signals the partner’s successful tying-up to the point of self-sacrifice, opposing and overcoming the results of all other Filter Tests. which would have let the antagonist prevail. This specific narrative simulation does not of course falsify the implications of the experimental literature, but illustrates how the interaction between AAs and RAs and the respective tests may lead to different outcomes from the ones expected on the basis of the ‘commonsense of human mating’—thus attaching to these apparently ‘anomalous’ cases a high informational value.

## 4. Results. *Jealousy Incarnate*: Can a Woman Love Two Men at Once?

### 4.1. The Narrative Context

The Female Lead (FL) of the story is a girl full of goodwill but with meager expectations of success, both professionally and sentimentally. Her lifetime dream is to become a news anchor, and she even managed to be hired by one of the main Korean TV networks, but only as the weather report girl, sent on air at the end of the news, for about 60 s daily. Pay is low and not enough to cover the financial needs of her younger student brother, but she does not get disheartened easily and takes extra gigs whenever available, striving for the acceptance and sympathy of others in a highly hierarchical, competitive job environment.

She does not look sophisticated but rather simple and genuine, she does not wear designer clothes, but however her style speaks of self-care and personality, turning even cheap and sometimes unlikely outfits into good looks. On the romantic side she has clear ideas, and at work she readily singles out the man she likes, the network’s main anchorman, the Male Lead (ML) of the drama. FL thus features a strange mix of ambition and humility. Her aspirations seem to aim high, without however bothering about her real chances. At the same time, she willingly accepts her subordinate position, accommodates the situation, and does her best even when her availability is exploited by superiors and fails to be appreciated by colleagues. To the external eye, FL’s naïve candor seems out of place, and her intentions opaque, so that she is easily blamed as shallow and clueless, or to the contrary as malicious and calculating. In fact, she tenaciously follows a personal code of conduct within her own value system and interests, mostly unconcerned with what others may think or insinuate. Unsurprisingly, her romantic life seems equally miserable: ML does not even notice her, and when everybody laughs at her, ridiculing her for that persistent infatuation, so apparent and absurd, he considers her little more than an annoying insect to chase away.

K-drama stories often deal with processes of personal change, and in this story the change is sparked by the friendship that connects ML to the Male Antagonist (MA), who from the first time he meets FL promptly realizes all her charm and remarkable potential, instantly reframing her personal qualities commonly seen as flaws as unique gifts, especially precious in an environment of ruthless, competitive careerists. MA is the first who gets tied-up, and the interest he shows for FL makes her suddenly visible to ML, who becomes jealous of FL’s attentions and competitive toward his richer, more handsome and masculine friend.

In fact, ML has already been in crisis for a while and things seem to turn from bad to worse. After having lost popularity due to a job scandal and having been downgraded from main anchor to foreign correspondent from Thailand for three years, once he eventually comes back to Seoul, he discovers he has cancer, requiring surgery and radiotherapy. The humiliation, adding up to the shock of his possible death, comes from the fact that his cancer is a typically feminine one, a nipple tumor which could bring him back into the gossip, definitely destroying his career as a public figure, in addition to his manly image and his attractiveness for women. FL is familiar with this illness, which already killed her granny first and then her mother. Destiny rules that she has to get surgery for a suspect mammal cyst the very same day ML has his own surgery, and that they end up sharing the same hospital room, as well as the secret on ML’s illness. FL takes care of ML, protects him from the scandal, altruistically supports him, and in so doing she finally manages to digest her own ruinous, unrequited love for ML, which lasted so long and, by acknowledging this, to bring it to a close for good.

However, ML’s attitude toward FL changes. He is upset that she does not seem so intrigued by him like she once was, ruminating over the dilemma that she’s just pretending and masquerading her interest toward him behind an emotional concern for his pitiful condition, or that instead such condition has caused him to lose his sexual attractiveness to her. Moreover, MA has started a tight courtship on FL, and ML has become aware of his weaker position against the rival, who is moreover his only and dearest friend. However, it would be a mistake to think that ML is eventually interested in FL just because of manly pride and rivalry. In fact, the longtime sympathy between the two friends allows ML to realize what MA sees in FL, and to desire it in turn. FL is passionate and sincere, generous and daring, affectionate and capable to love selflessly. Moreover, despite her fragility, she is tenacious, strong and hard to discourage. Now that a handsome, fascinating, wealthy, gentle young man is in love with her and courting her, she does not forget to help ML, supporting him without expecting anything back, despite being in difficulty herself, out of sheer empathy.

ML feels his life is on the brink of collapse, and she is the only one to reach out to him, whereas turning her back on him would have been more logical instead. The most cherished things in his life—friendship, love, health, career, family ties—have become mutually conflicting, excluding one another, and excluding himself in turn. Now that even his brother has died before they could sort out their misunderstandings, now that his illness has been diagnosed and the fight to survive it has started, ML feels that he has no choice but to reckon with his failure and to save what can be saved of his life, family, friendship, and, not last, love, even if this implies that it will be now him, and no longer FL, to experience a one-sided love, a unilateral TU. ML ties-up and consequently FL witnesses an abrupt change in her position, now finding herself in a love triangle with two men, once out of reach, who now compete for her, and moreover she faces the unbelievable opportunity to participate in an audition for new anchors, which would allow her to get promoted and fulfil her dream.

### 4.2. The Scene of the Kiss

What is interesting at this point is understanding who FL will choose between MA and ML, and why. In this regard, we analyze a single scene in the drama, the one where FL is kissed for the third time. FL has already kissed both, but this third kiss deliberately sanctions FL’s definitive TU to ML, consequently excluding MA from the possibility of getting FL’s F-TU instead. This scene is fundamental as it clearly shows the role and the relative weight of the different female tests (Filter Tests and Bio-sexual Compatibility Test), and which one among the two proves to be crucial in terms of the TU.

The scene takes place in the late afternoon, within a hospital branch that has become empty at that late hour, offering ML the possibility to go through the last radiotherapy evening session, before the department closes, thus guaranteeing him some privacy. FL has accompanied him, and waits for him out of the locker room as he is undressing to wear his gown. FL gets a message on her phone; it is MA. They have started dating and FL seems to have accepted his courtship. That same morning, the young woman went through the audition that, if successfully passed, would allow her to become a news anchorwoman as well. MA writes: “*Don’t you have something to say to me? I want to hear all news first, whether it’s good news or bad. You need to lean on me first, okay?*” What MA is asking for is exclusivity, that is, being the first in FL’s heart and thoughts.

FL reflects as she re-evokes the words ML said to her: “*Don’t be swayed… by me. Don’t be swayed*.” At this point, she stands up and steps toward the closed locker room door, saying to ML inside: “*I will… never let you sway me*.” And him, with a resigned voice: “*Okay*”. She goes on: “*I will never like you again*.” And him, sighing: “*Okay*”. However, FL insists, asking herself and asking him: “*Am I crazy?*”, as he tries to curb this dangerous attitude of hers, asking her to stop: “*That’s enough*”, he says to her. The intuition is right: FL enters the locker room, pushing aside the curtain that separates ML from the vestibule and showing him a drawing he knows well, as he kept it in his room as the only proof of his secret love for her. FL tells him: “*Even if you draw me 1000 of these 100 days, I won’t look back*” (that is, ‘I will not turn back and love you again’). “*You must think I’d fall for you if you sweet-talked me*”, she goes on saying while tearing the paper into pieces and dropping them on the ground, “*But I won’t*”. It’s a challenge! She is provoking him and he replies once more: “*Okay, so go.*” He has no intention to accept that challenge, thus he pushes her beyond the curtain and shuts it right after, turning his back, but she opens the curtain again and moves toward him, going on: “*You know what a great guy your friend is, right? I like him more. I like Jung Won more than he likes me*”. In fact Jung Won, AM, is deeply in love with her, but what FL actually means is that he is much worthier to her than she, with all her limitations, could possibly be worth to him. We are entering the field of Filter Tests that measure a partner’s salience, as proven by the row of AM’s virtues that FL is about to list:

“*He is much more well-mannered than you. He’s warmer, nicer, more considerate, better looking, wealthier, nicer to me, has a nicer voice, gentler, doesn’t pretend and is honest. He works hard, is comforting, manly and successful. He isn’t fickle or yap. He looks good when he opens his mouth and when he closes it. He has a broad chest and they [the nipples] aren’t lopsided. He’s warm and comforting, and makes me want to hug him. The opposite. The exact opposite is you. You know that, right?*”

ML’s Olympic calm starts to ripple as, hitting the locker with the back of his head, he replies: “*Yes. You’re right. Okay? Did you barge in here to say that?*”, and nodding in a small voice, his eyes fixed on her lips: “*Get out.*” He grabs her, but she resists, saying: “*I got you disqualified from the anchor audition*”. Once more he pushes her beyond the curtain: “*Get out*”. The curtain is shut again by him, and reopened again by her. FL is once more inside that cramped space, she has trespassed again the boundary of intimacy symbolized by the curtain, saying: “*I’m an evil witch*”.

MA has successfully passed all the key Filter Tests that exist: The one on personality and intelligence (“*He is much more well-mannered than you. He’s warmer, nicer, more considerate…nice to me… gentler, doesn’t pretend and is honest. He works hard, is comforting… He isn’t fickle or yap*”), the one on wealth and status (“[He is] *wealthier… and successful*”), on physical aspect (“[He is] *better looking… manly… He looks good when he opens his mouth and when he closes it. He has a broad chest and they* [the nipples] *aren’t lopsided*”), a reference to ML’s surgery that affected his chest and nipple to take the tumor away, whereas MA has a healthy body. There is even the test on conformity (“*I got you disqualified…*”). The term ‘warm’ is repeated a second time, but whereas the first time it points at personality, now the reference goes to the physical contact that she means as ‘comfortable’ (“*makes me want to hug him*”). In this latter case, it is not about a Filter Test, but hints at the good physical feel, as she seems to imply that the Bio-sexual Compatibility Test might have been successful, and certainly completed with the kiss that has kicked off their dating. This is a curious situation. ML, unlike MA, has clearly failed FL’s Filter Tests but has surely passed the Bio-sexual Compatibility Test, otherwise she would not be now in the men’s locker room alone with him, but most likely his rival as well has successfully passed the same, fundamental test for FL’s F-RA. FL is possibly not fully aware of what she’s doing, but she is actually suggesting ML the right timing that will allow him to beat his friend, not as to the overall result of the tests, but in terms of picking the right moment. What FL’s accusing words conceal is a suggestion about timeliness: If now he will kiss her, she will tie-up and he will have won her heart.

To be fair, it is not true that ML has failed all of the Filter Tests, and FL is aware of that even if ML does not realize it. She has been silent on this point, to give more momentum to her provocation. The Filter Test that ML has successfully passed is certainly the most important in view of the characteristics of FL’s F-AA: The Test of Moral Responsibility. ML has done the only thing he could have made at that point of the story to save what could be saved and give at least a meaning, an acknowledgement and a sense of direction to his feelings. He has sacrificed himself for her, giving up to the only possibility he had to take back control of his anchorman career, and thereby guaranteeing FL a place in the audition for future anchors. Among all Filter Tests, the Test on Moral Responsibility is the only one that depends on the TU and confirms its existence, and in this case the existence of the M-TU, in that only a strong, deep TU in a valuable individual leads to the altruism that makes such a big sacrifice possible, against one’s personal interest and dreams, to the only benefit of the loved one. FL has not only realized how hard ML’s sacrifice has been, but through it she got the certainty of the existence of ML’s M-TU. The fact that ML’s gesture has been concealed rather than exhibited proves its sincerity: Showing off the sacrifice would have been to the contrary a form of manipulation, possibly exerted by a non-tied-up subject willing to prevail upon his rival. ML’s capricious, odd behaviors did not imply then a simulation of a TU, fueled by the competitive jealousy that always linked him to his friend, as in a game of undisclosed rivalry that makes a counterpoint to their reciprocal affection and respect. ML is as tied-up as MA, has passed the Bio-sexual Compatibility Test as MA did, but unlike MA has given himself away for her despite his conviction that she was lost. This is the element that changes his position in FL’s heart, the reason why she suggests the right timing to him and lets him kiss her—a further confirmation that the F-TU originates in the sexual hemicycle as a consequence of an intense stimulation of the F-RA, despite being conditioned by the approval of the F-AA in the psycho-emotional hemicycle as a result of the success of at least a single, relevant Filter Test.

When FL claims to be an evil witch, she presents herself as the typical fairy tale character that breaks the rules and makes use of sorcery for suspicious reasons, that is, she is saying she will not be fair to her boyfriend and will give ML the chance to be equally evil. At this point, ML starts to realize and warns her: “*It’ll be dangerous if you stay*”. She then offers a clue, hinting at the result of the audition, that justifies her behavior and provides him with the entitlement he has gained by sacrificing himself for her: “*I passed. I wanted to tell you first*”—an entitlement she is meanwhile denying to MA. ML is close, staring at her lips and insists: “*I warned you that it’s dangerous*”, but FL keeps on insisting in turn—another little push—and repeats: “*Jung Won is a great guy. He’s a good man*”. Then him: “*I agree*”, and her: “*You are… a bad person*”. He makes another step toward her, he is really close now, and repeats: “*I agree*”. She insists: “*You are… a bad man*”, and him again: “*I agree*”. FL stands still, not escaping physical closeness: “*You are a bad friend*”. Now the invitation is really explicit and ML takes the initiative: he says “*I agree with that, too*” and finally starts to kiss her.

### 4.3. Reading Behind the Lines: Decoding the ‘Strategic’ Communication Between Partners Through the Tie-Up Theory

As a further support to the interpretive reading of the scene of the F-TU just analyzed, we now consider a dialogue between the two main characters that takes place during the subsequent narrative unfolding, and that offers a real ‘signature’ of the interaction dynamics between the respective AAs and RAs and the action of the corresponding tests, as ML strives to guess which one between him and MA has managed to prevail in FL’s RA. This dialogue allows us to appreciate in finer detail how the Tie-Up Theory allows us to decode the communicative exchange of the partners in its ‘strategic’ role for the purpose of the formation of a long-term couple.

PM: “*Do you really like Jung Won and me exactly the same?*”

PF: “*My gosh! When I said I had a crush on you four years ago, how nice would it have been if you had liked me back?*” [in Tie-Up Theory’s terms, she means that it would have been good to start a TU-C with him].

PF: “*I liked you so much for three years. Back then, you never once looked at me. You never said a kind word to me.*” [and this could have been the reason why, despite ML had passed the Bio-sexual Compatibility Test back then, FL did not tie-up].

PF: “*Why are you saying you like me now and making my head explode?*”

ML does not answer.

PF: “*It’s nicer to receive love than to give it*” [it is not nice to be in a one-sided TU because you don’t get any rewards].

PF: “*Life was less hard with someone who liked me*” [being in a TU-C with Jung Won, FL would receive her shots of direct and indirect rewards].

PF: “*I was so attracted to Jung Won*.” [Literally, MA has successfully passed the Bio-sexual Compatibility Test just like ML].

PF: “*Why did you have to do this to me now?*” [Because FL, remaining in the TU-C with MA, would have ended up tying-up to MA, and for ML there would have been no more chance to start his own TU-C with her].

PM: “*Do you like Jung Won more?*” [‘have you tied-up to Jung Won?’].

PF: “*I don’t know. I don’t know. I don’t know. I don’t know*.” [It’s FL’s F-AA that complains she is not receiving signals on this from her own F-RA].

PM: “*You like me more, right?*” [‘you have tied-up to me, right?’].

PF: “*My feelings for you are hidden way deep down inside. How would I know?*”

This seems a way not to respond to ML’s question, but in fact FL involuntarily leaks a detail that, in light of what we know about F-RAs, confirms that she is aware to have tied-up to ML. In this case, F-AA knows perfectly to whom the F-RA has tied-up, then FL is conscious about her own TU. The issue is that FL’s F-AA disagrees with her own F-RA and does not accept that TU which puts the AA into dissonance, especially because of her sense of guilt toward MA, a really good guy who also successfully passed the whole battery of tests, but that unfortunately did not guess out the timing that would have enabled him to be the first to cause a peak of excitement in FL’s F-RA. Thus, when the F-AA says that FL’s sentiments for ML will remain buried *in the deep*, she is actually saying that she will not give voice to the choice of the F-RA, which acts autonomously but below the threshold of consciousness, that is, in the deep. Thus, the F-AA will pretend to know nothing about her own F-RA.

PF: “*I thought there was nothing left, but it keeps popping up. I don’t even know how deep I locked it up. So how should I know whom I like?*” [The internal conflict, that is the discordance between F-AA and F-RA, is apparent. At this point, either the F-RA manages to take over the F-AA, or there is the need to act upon the F-AA to force it to accept the F-TU].

MA chooses to reinforce FL’s F-AA opposition to the F-TU for ML by proposing to FL to marry (and in the Far East, a marriage offer amounts to an additional Filter Test which, if the proposing partner deserves attention, provides a huge reward to any F-AA). To the contrary, ML chooses the strategy to reinforce the excitement of the F-RA, hoping for its eventual takeover of the F-AA, offering to FL sexual intimacy instead: “*Sleep with me!*”

## 5. Conclusions

At first glance, *Jealousy incarnate* might look like a classical example of romantic comedy tailored to a female audience willing to indulge in escapist fantasies: A young woman, FL, modest, disadvantaged, and without perspectives, who suddenly can choose between the man of her dreams (ML) and a ‘prince charming’ (MA) who is even more perfect and desirable than she could ever dream of. Seen from this angle, the issue of who is chosen and why appears pointless. However, in view of the story’s social cognition valence, this superficial depiction is easily turned down.

FL is not a Cinderella who ‘magically’ finds two desirable partners at her feet for some twist of fate. All the other characters are hostages of some form of jealousy (from which the title) fueling their desire to compete and excel upon others, but also to be the object of the others’ desire, to exploit it at their advantage and grab as many rewards and attention as possible to feed their ego. FL is the only character that proves to be immune from this jealousy fever, and this is the reason at the root of the deep psychological fascination, which the two male figures, ML and MA, end up feeling for her, turning their RAs on. FL’s non-competitive attitude could be seen as a sign of inadequacy and lack of self-confidence. However, the opposite is true: FL thinks big and is ambitious and daring, to the point of superficially appearing as a silly, naïve dreamer. She longs for a career and a man that are apparently unreachable, and she seems unwilling to rationally assess the feasibility of her plans, especially if related to her capacities and actual possibilities. Still, despite that, she does not surrender to jealousy and does not enter the competition. FL is in other words a representative example of a Quiet Ego [74], i.e., an ego focused upon eudemonic growth goals [75] transcending the mere pursuit of self-interest [76]. Tellingly, the developmental path of eudemonic well-being naturally lends itself to be conceptualized in terms of narrative identity [77], rooting its process of psychosocial integration upon the search for a shared existential meaning [78], conducted through a vigilant, sophisticated exploration of the developmental potential of interpersonal relationships [79], and an awareness of the transitions that punctuate personal growth paths leading to a socially mature personality [80].

FL does not competitively pit herself against others, nor she envies those who have what she wants: This allows her to persist in pursuing her goals without wasting energies, and to maintain her capacity to choose while eschewing the misleading influence of external circumstances and social pressures. FL is far from a clueless girl arbitrarily parachuted into a daydreaming situation. Quite the opposite, she deeply resonates with the RAs of the male characters who, for different reasons, have had enough of the competitive, opportunistic social world that surrounds them. Both ML and MA have passed the Bio-sexual Compatibility Test, and FL’s F-RA is therefore stimulated by both. From the viewpoint of the female tests, if both partners are physically attractive, the difference lies in the timing (who will excite the F-RA first, bringing it to a peak of indirect reward), and in the outcome of the Filter Tests. Generally, the Filter Tests, being an expression of various forms of social conditioning, privilege the most sought after positional resources: Status, wealth, physical prowess, social networks and approval, and even (real or simulated) evidence of the TU of the opposite-sex subject. However, for an uncompetitive FL, the Filter Test that eventually counts is the most unlikely and difficult to pass, the Test of Moral Responsibility, that becomes the crucial choice determinant and also the proof of the extraordinary personal transformation of ML, possibly the story’s most jealous and selfish character, who thanks to such transformation is saved from a likely, irreversible existential failure.

FL does not passively wait for the male subject with the best timeliness and initiative in involving her into a TU-C. She puts her F-AA into play, evaluating them also from the viewpoint of Filter Tests. This choice would seem to favor MA, who clearly triumphs in all the most popular tests (attractiveness, wealth, personality), as compared to ML, who has a bad character, is ill, and also has financial problems. However, despite that the success in the Filter Tests is generally evaluated cumulatively (the more tests are passed, the better), the relative weight of each test depends upon the F-AA’s value orientation. FL’s choice would seem straightforward, and in fact she accepts MA’s courtship, relegating ML into an affective relation of friendly camaraderie. Even ML’s extreme sacrifice, who renounces to his career progression to favor FL (a choice once unthinkable for a person like him, now uniquely determined by the strength of his TU) could look like a pleasant gift but certainly not a game changer for a competitive FL who is interested in extracting the biggest possible benefit from the co-occurrence of the M-TUs of the male rivals. What makes the difference in the choice are instead FL’s Quiet Ego and her personal integrity. For her, the weight of the Moral Responsibility Test prevails upon all other combined Filter Tests. The F-AA momentarily takes over and is unwilling to passively wait for the one who will successfully excite her F-RA first. FL decides for once to be unfair to MA, suggesting herself to ML the right timing to generate in her F-RA an exclusive F-TU for him. Can a woman love two men at once, then? In a wide sense, the answer is affirmative: A woman can ‘love’ two men insofar as the Bio-sexual Compatibility Test is successfully passed by two different candidates. However, in the end, she can only tie-up to one of them because the TU is exclusive: Once the RA reaches its excitement peak with an opposite-sex subject, it is incentivized to extract the highest possible reward from the interaction with that subject rather than risking to dissipate the rewards through explorative interactions with several different rivals, even if compatible. The passive nature of the RA implies that such excitement is not the consequence of a deliberate choice but reflects the intensity of the received stimuli. Like in evolutionary searches on fitness landscapes [81] such as e.g., in multi-armed bandit problems [82], the excitement peak for the RA has the function to prioritize exploitation over exploration, that is, to make the RA focus on the opposite-sex subject who rewarded it. If the two partners also manage to kick-off a TU-C, the self-catalytic nature of the cycle, through the sequence of mutually satisfactory direct and indirect rewards that each partner obtains from the other, will suitably amplify the rewards from the interaction, locking the partners into a stable couple. Even if two compatible rivals could therefore both be potentially able to successfully spark a TU-C with an opposite-sex subject, once one of them has excited the partner’s RA and a cycle has been launched, the other has little chance to reverse the trend. Thus, once the TU is formed and insofar as it remains solid, the RA focuses on the stimuli coming from the subject it has tied-up to. If for ‘love’ we mean a relationship founded upon a TU, then, the answer is negative: A woman can be tied-up to one man at a time only. In terms of the uniqueness of the TU, a similar argument also holds for men and their M-RAs—keeping in mind the different nature of the excitement of the RAs for women (bio-sexual) and men (psycho-emotional).

Despite the appearances, the mating dilemma analyzed in *Jealousy incarnate* is the opposite of an escapist fantasy. The social cognition valence of this narrative ‘simulation’ lies in the recognition that the super-cooperation between the partners that is at the root of a solid, vital TU-C depends upon the capacity to generate a flow of reciprocal rewards that is not conditional upon the instrumental benefit of the romantic relationship. This is essentially the evolutionary rationale of the TU: Creating a permanent disposition to reward the partner with the timing and under the forms required by the TU-C, to strengthen the couple bond according to a self-catalytic logic. With a FL such as that of *Jealousy incarnate*, ML loses to MA on the basis of every choice criterion but his own, one-sided and apparently hopeless, vow to sacrifice all for FL—even more striking if made by a character previously known for his arrogance, competitiveness, and selfishness, as well as a powerful illustration of the paradoxical nature of the process of self-actualization [83]. Self-actualization is what brings the main characters to transcend the opportunistic dimension of mating through the discovery of the transformational power of moral responsibility and self-sacrifice in the construction of a romantic couple bond, and the fictional setting enables us to appreciate and understand how this outcome is brought about by a subtle interplay of socio-psychological and biological forces. MA has immediately acknowledged FL’s qualities and has put all his psychological and social resources at her disposal, but ML, in the end, has done much more: He has let himself be transformed by his relationship with FL, accepting a very high existential risk without reasonable hopes for success, ending up publicly exposing all his weaknesses to protect her. To someone, this may be more valuable than a handsome, wealthy, caring partner.

Unlike the anonymous, more or less attractive men and women with more or less symmetrical faces or seductive postures whose photos are customarily shown to experimental subjects in lab environments, the characters of our story, and more generally of romantic stories with a significant social cognition valence, are ‘individuals’ with unique characteristics, which in best cases we will never forget. This is the power of narrative simulations: Allowing to experiment with lifelike situations without ethical concerns, to gather valuable experience that can be put to use in life situations. It is interesting that K-dramas, also outside of the romantic genre, are starting to be used as narrative simulation resources for intervention studies on social issues such as school bullying and mental health [84], and are being recognized as an emergent resource for the negotiation of new femininities in traditionally patriarchal socio-cultural environments [85]. To K-drama viewers, and more generally to audiences of socially validated romantic fictions, certain contents may guide personal experiences, not because viewers think that what they saw on screen is a ‘realistic’ depiction of life situations [86], but because it parsimoniously models key features of such situations that are generally difficult to recognize and interpret. For many young people exploring romantic relationships in social contexts marked by a complex interplay between custom and social conditioning vs. pursuit of intimacy and self-realization in mating choices, the social cognition valence of such stories offers a useful resource for critical reflection, and makes K-dramas so widely appreciated, as compared to other romantic popular culture narratives, sometimes also including traditionally more established Western ones. There is already a growing body of research on the local socio-behavioral effects of the global reception of romantic K-dramas, and we expect it to further develop in the next few years through an increasing number of country studies. Clearly, there are many other valuable sources of narrative social cognition of romantic relationships other than K-dramas, as documented in previous studies [28,36,37]. However, the fact that South Korea has been simultaneously experiencing both a crucial moment of socio-cultural transition with huge consequences on the social logic of personal relationships and mating choices, and a flourishing of its cultural industry, leading to a massive creative production exploring the dilemmas and complexities of romantic interactions—and making it available to a wide, culturally diverse global audience that constantly operates as a test bed for the social validation of narratives—makes of K-dramas an especially interesting source for future research. For this reason, we look forward to more studies on the social cognition valence of romantic K-dramas.
